# A Sialic Acid Binding Site in a Human Picornavirus

**DOI:** 10.1371/journal.ppat.1004401

**Published:** 2014-10-16

**Authors:** Georg Zocher, Nitesh Mistry, Martin Frank, Irmgard Hähnlein-Schick, Jens-Ola Ekström, Niklas Arnberg, Thilo Stehle

**Affiliations:** 1 Interfaculty Institute of Biochemistry, University Tübingen, Tübingen, Germany; 2 Division of Virology, Department of Clinical Microbiology, Umeå University, Umeå, Sweden; 3 Biognos AB, Göteborg, Sweden; 4 Department of Molecular Biology, Umeå University, Umeå, Sweden; 5 Laboratory for Molecular Infection Medicine (MIMS), Umeå University, Umeå, Sweden; 6 Department of Pediatrics, Vanderbilt University School of Medicine, Nashville, Tennessee, United States of America; Institut Pasteur, France

## Abstract

The picornaviruses coxsackievirus A24 variant (CVA24v) and enterovirus 70 (EV70) cause continued outbreaks and pandemics of acute hemorrhagic conjunctivitis (AHC), a highly contagious eye disease against which neither vaccines nor antiviral drugs are currently available. Moreover, these viruses can cause symptoms in the cornea, upper respiratory tract, and neurological impairments such as acute flaccid paralysis. EV70 and CVA24v are both known to use 5-*N*-acetylneuraminic acid (Neu5Ac) for cell attachment, thus providing a putative link between the glycan receptor specificity and cell tropism and disease. We report the structures of an intact human picornavirus in complex with a range of glycans terminating in Neu5Ac. We determined the structure of the CVA24v to 1.40 Å resolution, screened different glycans bearing Neu5Ac for CVA24v binding, and structurally characterized interactions with candidate glycan receptors. Biochemical studies verified the relevance of the binding site and demonstrated a preference of CVA24v for α2,6-linked glycans. This preference can be rationalized by molecular dynamics simulations that show that α2,6-linked glycans can establish more contacts with the viral capsid. Our results form an excellent platform for the design of antiviral compounds to prevent AHC.

## Author Summary

Coxsackievirus A24 variant (CVA24v) and enterovirus 70 (EV70) are responsible for several outbreaks of a highly contagious eye disease called acute hemorrhagic conjunctivitis (AHC). These viruses represent a limited set of human picornaviruses that use glycan receptors for cell attachment. Until now no data has been available about the binding site of these glycan receptors. We therefore determined the structure of the entire virus capsid in its unbound state and also together with several glycan receptor mimics and could establish the structure of the receptor binding site. CVA24v recognizes the receptor at a solvent exposed site on the virus shell by interactions with a single capsid protein VP1. Moreover, we identified a glycan motif favoured for CVA24v binding and confirmed this preference biochemically and by *in silico* simulations. Our results form a solid basis for structure-based development of drugs to treat CVA24v-caused AHC.

## Introduction

Coxsackievirus A24 variant and enterovirus 70, members of the Picornaviridae family, cause acute hemorrhagic conjunctivitis, a highly contagious eye infection [1], [2]. During the last decades CVA24v has been responsible for several outbreaks and two pandemics [3]–[9]. Besides hemorrhagic conjunctivitis, the two viruses can also cause symptoms in the cornea, upper respiratory tract, and neurological impairments such as acute flaccid paralysis [2], [5], [6], [10]. Despite the recurring appearance of AHC caused by CVA24v, to date, neither vaccines nor antiviral drugs are available for the prevention or the treatment of the disease.

Most picornaviruses engage protein receptors such as decay-accelerating factor (CD55, DAF) [11], [12], intercellular adhesion molecule 1 (ICAM-1) [13], the low-density lipoprotein receptor (LDL-R) [14], the coxsackie and adenovirus receptor (CAR) [15], and integrins [16], [17]. However, the AHC-causing human picornaviruses CVA24v and EV70 use glycan-containing receptors for cell attachment [1], [18]. Both viruses engage glycans that terminate in the sialic acid 5-*N*-acetyl-neuraminic acid (Neu5Ac). Cell binding and infection studies showed that EV70 binds Neu5Ac in the context of an α2,3 linkage [18], while CVA24v is able to use both α2,3- and α2,6-linked Neu5Ac as receptors, with some preference for the α2,6-linkage. The sialic acid-containing receptor is used by CVA24v on corneal but not conjunctival cells [19]. Until now, the molecular determinants underlying these interactions have been unknown for either virus.

## Results

### Crystal structure of the CVA24v particle

**Figure 1:**
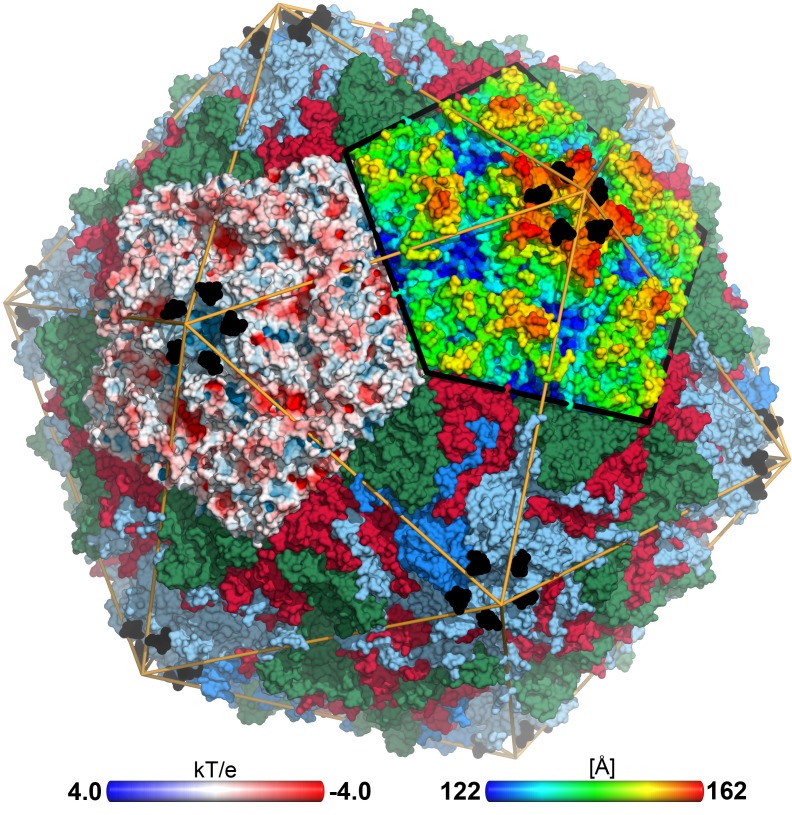
CVA24v in complex with its glycan receptor. The capsid structure of CVA24v with the capsid proteins VP1 (light blue), VP2 (green), VP3 (red) is shown in a surface representation. VP4 is located inside the capsid not visible in this figure. The Neu5Ac entity (black) is located at a positively charged, solvent exposed region of VP1. The atoms of one pentameric section (left) are colored according to the electrostatic potential using a color scale from red to blue. The adjacent pentameric section (right) was colored according to the distance from the center of the capsid, ranging from blue (122 Å) to red (162 Å).

To establish a structural basis for the recognition of sialic acid by CVA24v, we first determined the structure of intact CVA24v, an isolate of the Malaysia outbreak occurring during the 2002–2004 AHC pandemic [5], at 1.40 Å resolution (Table 1, Figure S1). As is typical for picornaviruses, the four capsid proteins (VP1-4) assemble into an icosahedral pseudo T3-capsid [12], [20], [21] (Figure 1). VP1 is close to a fivefold axis of the capsid; VP2, close to a twofold; and VP3, close to a threefold. VP4 lies inside the capsid and carries a myristyl group at its N-terminus. Picornaviruses have so-called non-polar pocket factors that modulate the interactions to receptors of the immunoglobulin superfamily, e.g ICAM-1 and DAF since they bind in regions that lie beneath the receptor binding site of the picornavirus capsid (so-called “canyon”) [22]. An unbiased (Fo-Fc)-omit map clearly reveals the presence of a branched, elongated pocket factor in CVA24v (Figure S2). However, the identity of this molecule remains unclear, perhaps due to multiple conformations or an inhomogeneous mixture of pocket factors present in the virion. A structural comparison [23] of ten different homologous picornavirus capsids (Figure S3, Tables S1 and S2) shows that CVA24v differs primarily at the *N*-terminal and *C*-terminal regions and at the solvent-exposed loops of the “jelly-roll” fold proteins VP1, VP2, and VP3. Compared to the structural homologues the most substantial structural differences are observed in the BC- and DE-loops of the CVA24v capsid.

**Table 1:** Data collection and refinement statistics^a^.

	CVA24v	CVA24v -6SL	CVA24v- DSLNT	CVA24v- acidic
**Data collection statistics**				
Resolution [Å]	29.9–1.40 (1.48–1.40)	50–1.70 (1.75–1.65)	50–1.90 (1.99–1.88)	99–2.90 (3.07–2.90)
Spacegroup	I222	I222	I222	P3_1_21
Unit cell [Å]	a = 306.56 b = 366.51 c = 368.10	a = 306.39 b = 366.10 c = 367.70	a = 304.48 b = 365.28 c = 366.47	a = 303.23 c = 763.61
No. of unique reflections	3666980 (401464)	2413465 (378574)	1552490 (261949)	826150 (98046)
R_meas_ [%]	9.6 (79.8)	15.5 (62.9)	17.3 (55.0)	15.5 (46.5)
CC(1/2)	99.8 (61.5)	99.2 (70.5)	98.9 (77.8)	99.3 (78.9)
Completeness [%]	92.1 (62.5)	99.2 (96.5)	95.4 (77.3)	92.7 (68.4)
Multiplicity	4.6 (3.3)	4.6 (3.5)	6.4 (3.0)	6.7 (2.2)
I/σ(I)	11.6 (1.6)	7.2 (1.7)	9.0 (2.0)	9.20 (2.0)
Wilson B [Å^2^]	18.3	20.6	16.6	49.0
**Refinement statistics**				
R_work_	14.9	14.9	17.7	18.9
rmsd bond length	0.004	0.006	0.006	0.005
rmsd bond angle	1.13	1.16	1.18	1.06
Ramachandran angles				
Favoured [%]	95.78	95.99	95.83	95.49
Outliers [%]	0.23	0.24	0.24	0.24

a Values for the highest resolution shell are shown in parenthesis.

### Glycan receptors for CVA24v binding

**Figure 2:**
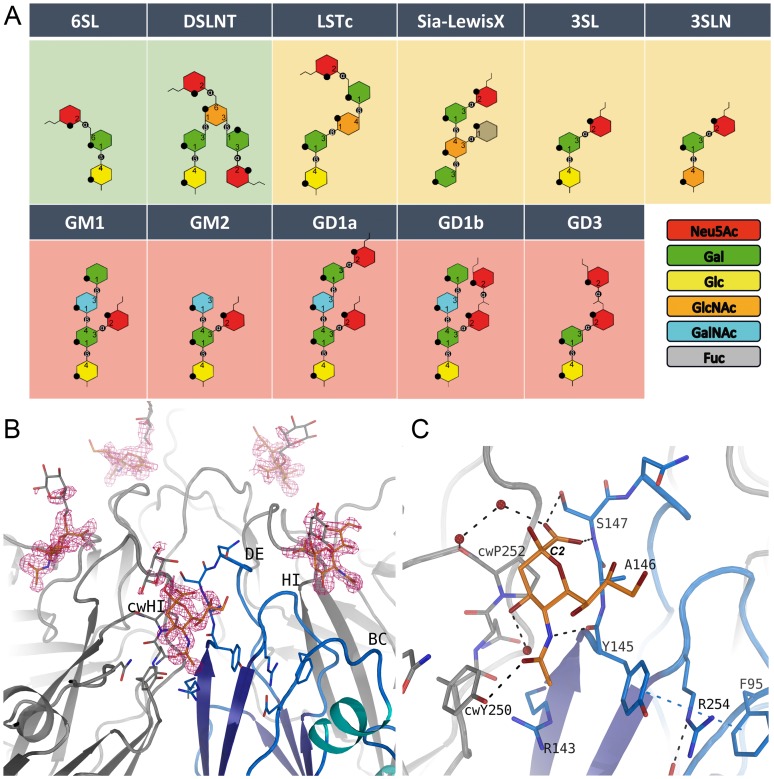
Glycan binding and attachment to CVA24v. (A) Overview of all glycans used in our incorporation experiment. The glycans 6SL and DSLNT bind well to CVA24v based on the electron density (green background). Very weak binding is observed for the LSTc, Sialyl-LewisX, 3SL and 3SLN (yellow background), and no binding could be detected for GM1, GM2, GD1a, GD1b, and GD3 (pink background). (B) The unbiased (Fo-Fc)-omit map (2.9σ, pink) revealed binding of the Neu5Ac entity (orange) of DSLNT and 6SL between two protomers with main interactions to the DE-loop and the HI-loop of clockwise rotated (cw) protomer. A galactose entity is shown (grey, not included into the deposited coordinates) which emphasize the direction of glycan binding towards the solvent. (C) Neu5Ac is recognized by hydrogen bonds to Y725, S727 and cwY830. The carbon atom C2 linking the adjacent glycan entity is marked.

Neu5Ac is required for infectivity of CVA24v; the roles of additional sugar moieties and the preferred Neu5Ac linkage have not been determined. In order to advance an understanding of the requirements for CVA24v binding to sialylated glycans, we derivatized CVA24v crystals with eleven physiologically relevant, commercially available sialyloligosaccharides (Figure 2A) that differ in glycan composition and linkage. We determined all structures to high resolution. In unbiased (2Fo-Fc)-omit maps, we observed a contoured electron density at a σ-level of 1.0 for the glycan only for α2,6-sialyllactose (6SL) and disialyllacto-n-tetraose (DSLNT) (Figure 2A), a hexasaccharide that carries an α2,3 and α2,6-linked Neu5Ac. In contrast, we observed very weak binding (corresponding to a σ–level of 0.7 in a (2Fo-Fc)-omit map) for α2,3-sialylated glycans, and no detectable binding for any β–branched or α2,8-α2,3-disialylated glycan. Thus, our analysis indicated that CVA24v preferentially engages glycans that contain α2,6-linked sialyloligosaccharide epitopes.

### The receptor binding site

Neu5Ac binds to VP1 near the fivefold axis, at a solvent exposed, protruding region of the virion (Figure 1). The shallow, positively charged binding site (Figure 1) is formed by the BC- (residues 95-99) and DE-loop (residues 145–151) of one VP1 monomer and the HI-loop (residues 247-254) of a clockwise (cw) rotated VP1 protomer (Figure 2B). All 60 Neu5Ac-binding sites in the pseudo T = 3 CVA24v particle are free of crystal contacts and feature unequivocally defined electron density (Figure 2B) for Neu5Ac in the complexes with 6SL (CVA24v-6SL) and DSLNT (CVA24v-DSLNT). We detected additional difference electron density towards the pentameric VP1 channel in all structures. This electron density does not result from glycan binding and is most likely a remainder of the virus preparation, as it is observed in a different crystal form (CVA24v-acidic, Table 1). Moreover, flexibility of the residues 147–150 might contribute to the observed electron density. Neu5Ac is bound with a set of hydrogen bonds to the side chains of S147 and cwY250. Additionally, two main chain interactions contribute to the glycan recognition. The nitrogen atom of S147 participates in binding to the carboxylic group, whereas the carbonyl oxygen of Y145 accepts a hydrogen bond from the acetamido -NH group of Neu5Ac. Moreover, two water-mediated hydrogen bonds are formed to the carbonyl oxygens of cwP252 and cwY250 (Figure 2C). Residues Y145, A146, and cwP252 also contribute hydrophobic contacts with non-polar portions of the receptor. An extensive π-π-stacking network involving the side chains of Y145, R254 and F95 appears relevant for Neu5Ac binding as this interaction stabilizes the conformation of Y145. A comparison of the observed interactions with other virus-sialic acid complexes shows that the Influenza A virus hemagglutinin, which has an entirely different fold, recognizes the Neu5Ac moiety [24] with a similar main chain interaction pattern [25]. The glycine-serine-motif of Influenza A virus hemagglutinin is substituted by an Y145-A146-S147 motif in CVA24v but remains functionally conserved (Figure S4). The carboxylate group of the sialic acid is fixed by hydrogen bonds from a side chain Oγ of a serine residue and a main chain NH, and the main-chain carbonyl of the adjacent residue accepts a hydrogen bond from the acetamido-NH of Neu5Ac. This situation is similar for influenza virus hemagglutinin (pdb-code: 1HGG), although the serine residue interacts with the carboxyl group from the opposite side.

Although the asymmetric unit of the crystals contains 15 crystallographically independent copies of the binding site, the electron density beyond Neu5Ac is not well defined in any of these sites in either structure. The Neu5Ac atom C2, to which additional sugars are attached in 6SL and DSLNT, projects away from the virion surface (Figure 2B). The electron density in the vicinity of the Neu5Ac C2 atom also leads towards the solvent, suggesting that additional carbohydrates attached to Neu5Ac project away from the virus, without engaging in significant contacts.

### Binding studies with glycan-incubated virions

**Figure 3:**
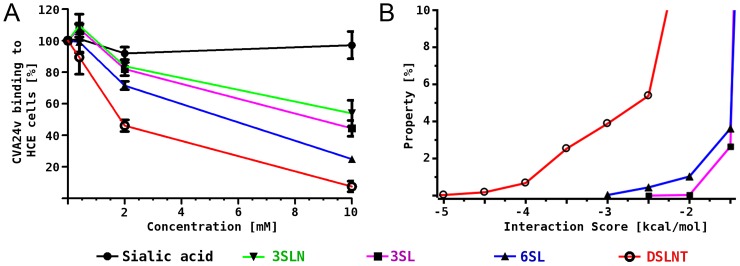
CVA24v binding inhibition assay and MD simulations. (A) ^35^S-labeled CVA24v virions showed substantially reduced binding to human corneal epithelial cells when the virions were pre-incubated with α2,6-linked glycans 6SL and DSLNT, while the effect on α2,3-linked glycans 3SL and 3SLN was less pronounced. (B) Histogram of calculated interaction scores confirmed the preference of α2,6-linked glycans and is in line with the structural and biochemical observations.

To complement the results from the structural analysis, we performed binding inhibition assays using ^35^S-labeled CVA24v virions that were pre-incubated with different glycans (Figure 3A). Compared with untreated virions, pre-incubation of the virus with DSLNT and 6SL substantially decreased the attachment to human corneal epithelial cells while α2,3-linked glycans such as 3SL and a2,3-sialyllactosamine (3SLN) had much lower effects, demonstrating the relevance of the observed interactions and specificity. A similar preference of α2,6-linked sialic acid glycans compared to 2,3-linked sialic acid compounds was very recently described in a glycan array analysis of human enterovirus 68 (EV68) [26]. The authors speculated that the observed preference for α2,6-linked sialic acid glycans in EV68 might result in an affinity for the upper respiratory tract.

### Binding studies *in silico*

In order to rationalize the differences in binding we investigated the molecular interactions between 3SL, 6SL and DSLNT and the CVA24v virion also *in silico*. A pre-generated conformational ensemble of each glycan was positioned into the binding site using Neu5Ac for superimposition. Histograms of an interaction score, which is based on AutoDock grid maps, were calculated (Figure 3B, for details see methods section). It was found that all three glycans can establish additional favourable contacts beyond Neu5Ac, which is shown by a significant population of conformations with negative interaction scores. The ranking is DSLNT> 6SL> 3SL, which is in good qualitative agreement with the competition experiments (Figure 3A). Additionally, we performed molecular dynamics simulations of the virus pentamer in complex with each of the three glycans in explicit solvent. A detailed intermolecular atom-atom contact analysis confirmed that DSLNT and 6SL can establish more favorable contacts than 3SL (Figure S5). These contacts are mainly transient, which is in excellent agreement with the observed lack of electron density beyond Neu5Ac.

## Discussion

We provide a structural basis for understanding the interactions of CVA24v with sialic acid-bearing glycan receptors at high resolution. Our data show that the preferred CVA24v receptor terminates in α2,6-linked Neu5Ac. Receptors terminating in α2,8-α2,3-disialylated glycans, such as GD1b and GD3, can clearly not engage CVA24v as these glycans would clash with protein residues when superimposing them onto the Neu5Ac entity, irrespective of which of the two Neu5Ac residues in GD3 of GD1b is used (Figures S6A and S6B). Moreover, steric restraints appear to interfere with the binding of β–branched α2,3-sialylated glycans (GM1, GM2, GD1a) to the virus, in line with our observation that these compounds do not bind CVA24v (Figures S6C and S6D), although MD simulations indicated that binding of GM1 to the virus seems possible. Finally, linear α2,3-linked sialyloligosaccharides such as 3SL bind less well to the virus and are also less efficient in blocking virus binding than their α2,6-linked counterparts. The crystal structures alone do not offer a straightforward explanation as only the Neu5Ac moiety is clearly visible in the electron density maps, and the CVA24v binding site could accommodate a range of glycan structures terminating in either α2,6 and α2,3 linked Neu5Ac. Sabesan and coworkers have reported a higher flexibility of α2,6-linked sialyloligosaccharides compared to their α2,3-linked counterparts [27], and our molecular dynamics simulations demonstrate that the increased flexibility of α2,6-linked glycans (6SL and DSLNT) yields a larger number of virus to receptor interactions and thereby likely favors the binding to α2,6-linked glycans. It is clear that the energetic differences are very subtle, which is reflected in our experiments that show weak binding to α2,3-linked glycans at the same binding site. However, it is important to bear in mind that the virus can simultaneously engage many glycans, and a small energetic difference in each binding site is therefore amplified in a cellular setting.

It is remarkable that CVA24v binds Neu5Ac in a surface-exposed, protruding region that appears to be an easy target for a neutralizing antibody response. The CVA24v binding site differs strikingly from the canyon-like areas that engage DAF [12], CAR [15] and ICAM-1 [13] in other picornaviruses. These deeply recessed “canyons” are thought to engage receptors in regions that are shielded from immune surveillance, and that can accommodate slender protein receptors [28]. However, not all picornaviruses engage their receptors via canyons. The LDL receptor binding site on human rhinovirus 2 (HRV2) [14] is located near the five-fold symmetry axes and does not bind to the canyon perhaps since this receptor is larger and would not fit into the narrow canyon structure. A closer look on the structure reveals that the LDL receptor binding site is located at the same position as the glycan binding site of CVA24v utilizing the BC-, DE-, HI-loop for recognition. LDL receptor binding to CVA24v is unlikely on the basis of a structural based sequence analysis, as the sequence differs substantially in these areas. Moreover, the BC-loop of CVA24v is significantly larger forming a rigid α-helix that would interfere with LDL receptor binding. While CVA24v represents the first structure of a human picornavirus bound to a glycan receptor, two animal picornaviruses, persistent Theiler's Virus DA strain [29] and a cell-culture adapted foot-and-mouth disease virus (FMDV) [20], [30], have been shown to bind glycans at the interface between VP1 and VP2 and the C-terminus of VP3. Although also solvent-exposed, this area is distant from the sialic acid binding site in CVA24v (Figure S7). Since none of the glycan receptors in picornaviruses target canyon residues, it seems that the principles that guide the engagement of picornaviruses with glycans might differ from those that underlie protein receptor binding. Glycan receptors such as sialylated oligosaccharides and glycosaminoglycans (GAGs) are conformationally flexible and negatively charged, and offer almost no options for hydrophobic contact formation, in contrast to protein receptors. In agreement with this observation, a survey of available virus-sialic acid complexes shows that they typically bind to shallow, surface-exposed regions of the capsid proteins [24].

It is tempting to speculate that the two picornaviruses that cause AHC engage related sialyated glycans that are expressed on ocular cells, thus linking their receptor binding specificity to tropism. To date, limited data are available about the glycan composition on such cells. Analysis of mucin-type *O*-glycans showed a highly unequal distribution of α2,6- and α2,3-linked sialylated glycans in tear films (48% and 8%, respectively), which contrasts with the distribution on conjunctival epithelial cells (3% and 47%, respectively) [31]. Based on our analysis, we would predict that CVA24v recognizes an easily accessible, unbranched α2,6-linked sialylated glycan motif on target cells, and that the high content of α2,6-linked sialylated glycan in tear films might facilitate virus spread within the eye.

CVA24v, EV70 and EV68 are the only human picornaviruses that use sialic acid-based receptors. The unique character of the CVA24v glycan binding site is revealed by a structure-based sequence comparison (Figure S8). The sequences that contribute to the Neu5Ac binding site shape a binding pocket not found in other CV strains, even if we take into account a replacement of amino acids that would functionally retain the sialic acid binding site. This analysis would exclude a similar binding of the same glycan receptor. Although EV70 binds α2,3-linked sialic acids [18], its sequence differs profoundly from that of CVA24v, including the CVA24v receptor binding region (Figure S9). We therefore expect that EV70 binds sialic acids in a mode that is distinct from the one observed here, which explains at least in part why CVA24v-caused infections more commonly includes respiratory symptoms than EV70-caused infections [1].

An anti-viral strategy identified by our results could target cell entry. Such an approach has been employed recently to develop potent inhibitors for ocular virus Adenovirus type 37 [32], [33]. A potential inhibitor of CVA24v could likewise consist of multivalent α2,6-linked Neu5Ac entities that in this case occupy the pentameric glycan binding site with substantial higher affinity, thereby blocking the cell attachment of the virus. A high affinity attachment caused by multivalent glycans has also been shown by Kitov and coworkers [34], and their pentameric STARFISH design of the Shiga-like toxin inhibitor might serve as template for the future development of drugs to treat AHC. Since the sialic acid binding sites cluster near the five-fold symmetry axis of CVA24v, such a molecule could still be similarly small and useful for topical applications.

## Materials and Methods

### CVA24v genome sequencing

Total RNA was extracted using Aurum Total RNA Mini Kit (BioRad) and cDNA was generated with Superscript III (Life Technologies/Invitrogen). The sequence was determined twice using dideoxy sequencing of PCR-amplified cDNA, each time on independent PCR products. The CVA24v genome sequence was deposited to the GenBank of the National Center for Biotechnology (http://www.ncbi.nlm.nih.gov/genbank/) with accession code KF725085.

### Sample preparation

The CVA24v strain (110390) used in this work originate from Malaysia and was isolated during the 2002–2004 pandemic [5]. ^35^S-labeled CVA24v virions were generated as previously described [1]. Briefly, normal human conjunctival (NHC) cells [35] were infected with CVA24v in serumfree medium containing Dulbecco's modified Eagle's medium (DMEM; Sigma-Aldrich), HEPES (pH 7.4; EuroClone, Milan, Italy), and penicillin-streptomycin (PEST; Gibco, Carlsbad, CA) with gentle agitation for one hour at 37°C. Cells were washed with phosphate-buffered saline (PBS; Medicago AB, Uppsala, Sweden) to remove non-bound virions and then starved of methionine and cysteine in Met/Cys-free medium (Sigma-Aldrich). After three hours, 35S-Met/Cys mixture (NEG-772 Easytag express protein-labeling mix; Perkin-Elmer, Wellesley, MA) and 1% fetal calf serum (FCS; Sigma-Aldrich) were added to the cells. Thirty hours after infection, Triton X-100 (Sigma-Aldrich) was added to a final concentration of 0.5% and centrifuged for 15 min at 3,000×*g*. Sodium dodecyl sulfate (VWR, Leicestershire, United Kingdom) was mixed with the supernatant to a final concentration of 0.5% and the mixture was laid onto a 30% sucrose solution and centrifuged for 3 h at 113,000×*g* at 18°C. The pellets were dissolved in 2 ml of 10 mM Tris-HCl, pH 7.5, and sonicated for 20 s. The mixture was loaded onto a discontinuous gradient of 1.2 and 1.4 g/ml CsCl and centrifuged at 107,000×*g* for 17 h at 4°C. The virion band was harvested and desalted on a NAP-10 column (Amersham Biosciences, Uppsala, Sweden) and stored in Tris-buffered saline (TBS) with 10% glycerol at −80°C. To produce non-labeled CVA24v virions, the ^35^S-Met/Cys-labeling step was neglected from the process described above. To concentrate the non-labeled virions for crystallization, 6 mg virions were laid onto a 20% sucrose solution and centrifuged for 3 h at 113,000×*g* at 18°C. The pellet was dissolved in 0.6 ml of TBS 10% glycerol to a final concentration of 10 mg/ml. The virions were stored in −80°C until use.

### Crystallization and derivatization

Crystallization screening was performed at 4°C by hanging drop vapor diffusion on siliconized cover slides. Therefore, the virus solution (10 mg/mL, 1 µL) was mixed with the crystallization buffer-I (200 mM Magnesium chloride, 3.4 M 1,6-Hexanediol, 100 mM HEPES pH 7.5) in a 1:1 ratio and placed over the reservoir solution. Rod-like crystals appeared after four days and grew to the final size of up to 80×80×300 µm^3^ within three weeks. To identify the glycan binding site of CVA24, we tested 6SL (15 mM), 3SL (15 mM), 3SLN (15 mM), LSTc (15 mM), Gd1a (15 mM), Sialyl-LewisX (15 mM), Gd1b (8 mM), DSLNT (8 mM), GM1 (8 mM), GM2 (8 mM), and GD3 (8 mM) for binding (Figure 2A). Therefore, the crystals were incubated in the glycan-containing solution for 1 h at 4°C, harvested and stored in liquid nitrogen until data collection. The same method was applied to obtain trigonal bipyramidal crystals. These crystals were obtained in acidic crystallization buffer-II (200 mM Calcium chloride, 20% (v/v) 2-Propanol, 100 mM Sodium acetate trihydrate pH 4.5) and grew to a final size of 150×150×250 µm^3^ within several days.

### Data collection and structure determination

Data collection was performed the beamline I03 at the Diamond Light Source in Didcot, UK. Special care has to be taken to avoid spot overlapping. Data were reduced by the XDS/XSCALE package [36]. All data sets were scaled to the native data set as reference. We obtained a data set of the native virus to a resolution of 1.40 Å and bound to 6SL and DSLNT diffracting up to at least 1.96 Å resolution. All crystals resulting from crystallization buffer-I are of orthorhombic spacegroup I222 containing two virus particles in the unit cell. We used a CHAINSAW [37] modified model of coxsackievirus B3 [12] as search model. This template structure was placed into the unit cell of the search model. The capsid of the template model was constructed from the NCS rotation translation matrices and the center of mass was calculated by a PYTHON script using PYMOL [38] modules to obtain the vector (t1 = 148.32 Å, −85.63 Å, −271.07 Å) to translate the template structure into the origin of the orthorhombic unit cell. Next, the template capsid was rotated around the x-axis by ε = 20.905° to orient the icosahedral two-fold axes of the virus capsid along the orthorhombic unit cell axes a, b and c. Finally, the template structure was translated into the center of the unit cell (t2 =  *a*/2, *b*/2, *c*/2) and the asymmetric unit was generated to include 15 copies of VP1, VP2, VP3, and VP4 using the NCS-operators of the search model. Initial phases were established by rigid body refinement procedure as implemented in REFMAC5 [39] with the pre-oriented asymmetric unit of the search model followed by a simulated annealing approach using PHENIX [40]. This approach yielded an R-factor of approximately 41%. Strict NCS-parameterization was applied. Several cycles of manually model correction with COOT [41] and refinement using REFMAC5 completed the model. Water molecules were placed using the COOT:find_waters algorithm and manually checked. Finally, the ligand molecules were placed into the unbiased (F_o_-F_c_)-difference omit map. All structure models were refined to possess excellent geometry with R-factors below 16% (Table 1). Figures were generated with PYMOL [38]. The electrostatic potential was mapped onto the surface by the use of APBS [42].

### Binding studies

^35^S-labeled CVA24v virions were used as previously described [1]. Briefly, 5000 ^35^S-labeled CVA24v virions/cell were incubated with or without different concentrations of glycans (6SL, 3SL, 3SLN, DSLNT; Carbosynth, Berkshire, United Kindom) (*N*-acetylneuraminic acid (sialic acid); Dextra, Reading, United Kindom) at 4°C, diluted in 50 µl of binding buffer (BB) containing DMEM, HEPES, and 1% bovine serum albumin (BSA; Roche, Stockholm, Sweden) for one hour with gentle agitation. Meanwhile, adherent human corneal cells (HCE cells) [7] were washed and detached with PBS containing 0.05% EDTA (PBS-EDTA; Merck, Darmstadt, Germany). The cells were recovered in growth medium (50% DMEM, 50% HAMs-F12, 1 mg/l human insulin, 100 µg/l cholera toxin, 2 µg/l human epidermal growth factor, 5 mg/l hydrocortisone, 10% FCS (all from Sigma-Aldrich), 20 mM HEPES and PEST) at 37°C with agitation. After one hour, 1×10^5^ cells/sample were washed with BB and added to the pre-incubated glycan-virion mixture. After 1 h of incubation at 4°C, cells were washed with BB to remove non-bound virions before the radioactivity of the cells was measured using a Wallac 1409 scintillation counter (Perkin-Elmer, Waltham, MA).

### Molecular modelling

Conformational ensembles of 3SL, 6SL and DSLNT were derived from 100 ns molecular dynamics simulation at 310 K using TINKER (http://dasher.wustl.edu/tinker/). The MM3 force field [43] and a dielectric constant of 4 were applied. The size of each ensemble was 100000 frames. Affinity grids for the virus receptor were calculated using AutoDockTools (http://mgltools.scripps.edu) and the *autogrid* program of AutoDock 3.05 [44]. All further processing was performed using Conformational Analysis Tools (CAT) (http://www.md-simulations.de/CAT/). The conformational ensembles were positioned into the binding site of the crystal structure using Neu5Ac for superimposition. For DSLNT the α2,6-linked Neu5Ac was positioned into the binding site. Gasteiger atom charges were assigned to the glycan atoms by CAT using OpenBabel (http://openbabel.org). Interaction scores for each frame were determined from the AutoDock affinity grids by using all glycan atoms except the atoms of Neu5Ac.

Explicit solvent molecular dynamics simulations of the virus pentamer in complex with 3SL, 6SL and DSLNT were performed at 310 K using YASARA [45]. AMBER03 [46] forcefield was used for the protein and GLYCAM [47] for the carbohydrates. Different representative glycan conformations were positioned into each of the five binding sites of the virus pentamer using CAT. 20 ns were sampled for complexes of 3SL and 6SL and 30 ns for DSLNT. Atom-atom contact analysis was performed using CAT. Simple atom-atom distance criteria were used for counting favourable interactions (hydrophobic: C-C distance <4.0 Å; H-Bond: donor-acceptor distance <3.5 Å).

### Accession numbers

Structure factors and atomic coordinates have been deposited in the Protein Data Bank (rcsb.org) with accession codes 4Q4V, 4Q4W, 4Q4X, and 4Q4Y.

## Supporting Information

Figure S1 **Stereo representation of the electron density around the sialic acid binding site.** A (2Fo-Fc)-electron density map around the glycan binding site is shown in a stereo representation to elucidate the quality of the refined CVA24v-6SL model. The sialic acid entity (light orange), the VP1 protomer (grey) and the clockwise rotated VP1 protomer (cyan) are shown in a stick representation. Water molecules (red) are shown as spheres. Some residues (727-730) in the DE-loop of VP1 showed a higher flexibility (not shown) which is reflected in higher B-Factors (26 Å^2^ compared to the mean of 13 Å^2^ for all protein atoms).
(TIF)

Figure S2 **Electron density for the pocket factor in CVA24v.** The protomers VP1 (blue), VP2 (green) and VP3 (red) are shown around the pocket factor cavity. The pocket factor, a hydrophobic compound bound in a cavity on the bottom of the canyon of the virus [22] is important for virus stabilization and is released during the attachment of immunoglobulin-like receptors at the canyon to facilitated RNA release. We found substantial positive (Fo-Fc)-electron density (orange, σ-level of 2.9) in this cavity of CVA24v for a branched ligand. A ceramide would in principle fit into the electron density but could not unambiguously identify as a pocket factor. A superimposition with coxsackievirus B3 (CVB3) (4GB3, black) revealed differences in the pocket that would exclude the binding of a long fatty acid as observed for this strain.
(TIF)

Figure S3 **Assembly of the capsid proteins and structure comparison.** The capsid proteins VP1 (blue), VP2 (green), VP3 (red), and VP4 (yellow) are shown as cartoon and as surface representation from the identical orientation. The termini of the protein chain are labeled. Moreover, the capsid proteins VP1-3 were colored by the mean Cα rms deviation of ten virus capsid proteins listed in table S2 from blue (zero rms deviation) to red (4.2 Å rms deviation). A close-up of the glycan binding region (black box) revealed the largest structural differences in the DE- and BC- loop.
(TIF)

Figure S4 **Similarities in sialic acid recognition between CVA24v and influenza virus hemagglutinin.** A superposition of the sialic acid-binding regions is shown, with the sialic acids colored orange for CVA24v and black for influenza hemagglutinin (pdb-code: 1HGG). The main chain interaction of CVA24v and influenza virus hemagglutinin to Neu5Ac are generally similar, although serine interacts with the carboxy moiety from the opposite side.
(TIF)

Figure S5 **Trajectories of favourable intermolecular contacts.** Analysis of favourable (hydrophopic and H-bond) intermolecular atom contacts using Conformational Analysis Tools (www.md-simulations.de/CAT/). The molecular dynamics simulation was performed with the virus pentamer in complex with five ligands in explicit solvent. Interaction group trajectories are shown for one selected ligand only. A new interaction group (atom pair) is allocated if one of the following conditions were met: C-C distance <4.0 Å (hydrophobic) or H-bond donor-acceptor distance <3.5 Å. The Neu5Ac residue located in the crystallographically determined binding site was excluded from the analysis. It can be seen that the number of possible additional contacts increases in the sequence 3SL<6SL<DSLNT and that contacts are generally transient.
(TIF)

Figure S6 **Sterical clashes of glycans superposed to the Neu5Ac entity in CVA24v.** α2,8-Neu5Ac-α2,3-Neu5Ac linked glycans are unlikely to bind to CVA24v as binding would result in a collision with the protein skeleton (shown as pink spheres) independently of whether binding occurs with the bridged Neu5Ac entity (A) or terminal sialic acid entity (B). Moreover, steric restriction hampered binding of glycans with a β-branch in respect to the Neu5Ac moiety, as shown for GM1 (C) or GD1a (D).
(TIF)

Figure S7 **Location of picornavirus receptors.** Receptors (purple) of the immunoglobulin superfamily bind into the “canyon” of the picornavirus capsid as shown for coxsackievirus B3 in complex with CAR (A) (adopted from pdb 1JEW [15]) or coxsackievirus A21 in complex ICAM-1 (B) (adopted from pdb 1Z7Z [13]). Receptors that do not belong to this superfamily do bind elsewhere, e.g. Rhinovirus 2 in complex with the LDL receptor (C) (adopted from pdb 1V9U [14]) or glycan receptors (D) such as the oligosaccharide receptor (purple, labeled as 2) of Foot-and-mouth disease virus serotype A10_61_ (adopted from pdb 1ZBA [30]). The binding site of the glycan receptor (black) of CVA24v (labeled as 1) is located at the LDL receptor binding site of Rhinovirus 2.
(TIF)

Figure S8 **The unique binding site of CVA24v.** Important residues for Neu5Ac binding (marked yellow) are found on the BC-, DE-, and the HI-loop. We suggest that sialic acid binds (three red stars) only if the residues are functionally conserved in all three loop regions. A residue is concerned as functionally conserved, if its side chain is capable to perform a similar interaction, e.g. hydrogen bond compared to CVA24v. A sequence comparison revealed unique character of the glycan binding site of CVA24v as compared to other coxsackieviruses in species Enterovirus A, B, and C.
(TIF)

Figure S9 **Sequence comparison of the AHC-causing viruses CVA24v and EV70.** This figure was modified from ESPRIPT [48] output. α-helices, 3_10_-helices and π-helices are displayed as squiggles. The η and α symbol refers to a 3_10_-helix and α-helix, respectively. β-strands are rendered as arrows, strict β-turns as **TT** letters and strict α-turns as **TTT**. Strictly conserved residues are marked by a red box and similar residues are shown as red character. Positions of CVA24v involved in glycan recognition are marked by green spheres. The β-strands of the jelly roll motif are labeled from “B to I” in agreement with the jelly roll nomenclature.
(TIF)

Table S1 **Structurally related capsid proteins of VP1-3.^a^** Results of the structure comparison of VP1, VP2 and VP3 to ten different homologous picornavirus capsids using DALI [23].
(PDF)

Table S2 **Structurally related capsid proteins of VP4.^a^** Results of the structure comparison of VP4 to homologous picornavirus capsids using DALI [23].
(PDF)

## References

[1] Nilsson EC, Jamshidi F, Johansson SM, Oberste MS, Arnberg N (2008) Sialic acid is a cellular receptor for coxsackievirus A24 variant, an emerging virus with pandemic potential. J Virol 82: 3061–3068. 18184708 10.1128/JVI.02470-07 PMC2259016

[2] Wright PW, Strauss GH, Langford MP (1992) Acute hemorrhagic conjunctivitis. Am Fam Physician 45: 173–178. 1309404

[3] Aubry C, Gautret P, Nougairede A, Dussouil AS, Botelho-Nevers E, et al. (2012) 2012 outbreak of acute haemorrhagic conjunctivitis in Indian Ocean Islands: identification of Coxsackievirus A24 in a returned traveller. Euro Surveill 17: : pii = 20185. 10.2807/ese.17.22.20185-en 22687914

[4] Cabrerizo M, Echevarria JE, Otero A, Lucas P, Trallero G (2008) Molecular characterization of a coxsackievirus A24 variant that caused an outbreak of acute haemorrhagic conjunctivitis in Spain, 2004. J Clin Virol 43: 323–327. 18786853 10.1016/j.jcv.2008.07.017

[5] Ghazali O, Chua KB, Ng KP, Hooi PS, Pallansch MA, et al (2003) An outbreak of acute haemorrhagic conjunctivitis in Melaka, Malaysia. Singapore Med J 44: 511–516. 15024454

[6] Kuo PC, Lin JY, Chen LC, Fang YT, Cheng YC, et al (2010) Molecular and immunocytochemical identification of coxsackievirus A-24 variant from the acute haemorrhagic conjunctivitis outbreak in Taiwan in 2007. Eye (Lond) 24: 131–136. 19218990 10.1038/eye.2009.8

[7] Likar M, Talanyi-Pfeifer L, Marin J (1975) An outbreak of acute hemorrhagic conjunctivitis in Yugoslavia in 1973. Pathol Microbiol (Basel) 42: 29–35. 10.1159/000162718 239379

[8] Moura FE, Ribeiro DC, Gurgel N, da Silva Mendes AC, Tavares FN, et al (2006) Acute haemorrhagic conjunctivitis outbreak in the city of Fortaleza, northeast Brazil. Br J Ophthalmol 90: 1091–1093. 16809381 10.1136/bjo.2006.098822 PMC1857380

[9] Triki H, Rezig D, Bahri O, Ben Ayed N, Ben Yahia A, et al (2007) Molecular characterisation of a coxsackievirus A24 that caused an outbreak of acute haemorrhagic conjunctivitis, Tunisia 2003. Clin Microbiol Infect 13: 176–182. 17328730 10.1111/j.1469-0691.2006.01618.x

[10] Baidya BK, Basu RN, Chakraborty AK (1983) Recent epidemic of acute haemorrhagic conjunctivitis in Calcutta. Indian J Ophthalmol 31: 632–634. 6671779

[11] Plevka P, Hafenstein S, Harris KG, Cifuente JO, Zhang Y, et al (2010) Interaction of decay-accelerating factor with echovirus 7. J Virol 84: 12665–12674. 20881044 10.1128/JVI.00837-10 PMC3004353

[12] Yoder JD, Cifuente JO, Pan J, Bergelson JM, Hafenstein S (2012) The crystal structure of a coxsackievirus B3-RD variant and a refined 9-angstrom cryo-electron microscopy reconstruction of the virus complexed with decay-accelerating factor (DAF) provide a new footprint of DAF on the virus surface. J Virol 86: 12571–12581. 22973031 10.1128/JVI.01592-12 PMC3497627

[13] Xiao C, Bator-Kelly CM, Rieder E, Chipman PR, Craig A, et al (2005) The crystal structure of coxsackievirus A21 and its interaction with ICAM-1. Structure 13: 1019–1033. 16004874 10.1016/j.str.2005.04.011

[14] Verdaguer N, Fita I, Reithmayer M, Moser R, Blaas D (2004) X-ray structure of a minor group human rhinovirus bound to a fragment of its cellular receptor protein. Nat Struct Mol Biol 11: 429–434. 15064754 10.1038/nsmb753

[15] He Y, Chipman PR, Howitt J, Bator CM, Whitt MA, et al (2001) Interaction of coxsackievirus B3 with the full length coxsackievirus-adenovirus receptor. Nat Struct Biol 8: 874–878. 11573093 10.1038/nsb1001-874 PMC4152846

[16] Bergelson JM, Shepley MP, Chan BM, Hemler ME, Finberg RW (1992) Identification of the integrin VLA-2 as a receptor for echovirus 1. Science 255: 1718–1720. 1553561 10.1126/science.1553561

[17] Berinstein A, Roivainen M, Hovi T, Mason PW, Baxt B (1995) Antibodies to the vitronectin receptor (integrin alpha V beta 3) inhibit binding and infection of foot-and-mouth disease virus to cultured cells. J Virol 69: 2664–2666. 7533862 10.1128/jvi.69.4.2664-2666.1995 PMC188951

[18] Nokhbeh MR, Hazra S, Alexander DA, Khan A, McAllister M, et al (2005) Enterovirus 70 binds to different glycoconjugates containing alpha2,3-linked sialic acid on different cell lines. J Virol 79: 7087–7094. 15890948 10.1128/JVI.79.11.7087-7094.2005 PMC1112099

[19] Mistry N, Inoue H, Jamshidi F, Storm RJ, Oberste MS, et al (2011) Coxsackievirus A24 variant uses sialic acid-containing O-linked glycoconjugates as cellular receptors on human ocular cells. J Virol 85: 11283–11290. 21880775 10.1128/JVI.05597-11 PMC3194947

[20] Fry EE, Lea SM, Jackson T, Newman JW, Ellard FM, et al (1999) The structure and function of a foot-and-mouth disease virus-oligosaccharide receptor complex. EMBO J 18: 543–554. 9927414 10.1093/emboj/18.3.543 PMC1171147

[21] Plevka P, Perera R, Cardosa J, Kuhn RJ, Rossmann MG (2012) Crystal structure of human enterovirus 71. Science 336: 1274. 22383808 10.1126/science.1218713 PMC3448362

[22] Rossmann MG, He Y, Kuhn RJ (2002) Picornavirus-receptor interactions. Trends Microbiol 10: 324–331. 12110211 10.1016/s0966-842x(02)02383-1

[23] Holm L, Sander C (1995) Dali: a network tool for protein structure comparison. Trends Biochem Sci 20: 478–480. 8578593 10.1016/s0968-0004(00)89105-7

[24] Neu U, Bauer J, Stehle T (2011) Viruses and sialic acids: rules of engagement. Curr Opin Struct Biol 21: 610–618. 21917445 10.1016/j.sbi.2011.08.009 PMC3189341

[25] Sauter NK, Hanson JE, Glick GD, Brown JH, Crowther RL, et al (1992) Binding of influenza virus hemagglutinin to analogs of its cell-surface receptor, sialic acid: analysis by proton nuclear magnetic resonance spectroscopy and X-ray crystallography. Biochemistry 31: 9609–9621. 1327122 10.1021/bi00155a013

[26] Imamura T, Okamoto M, Nakakita S, Suzuki A, Saito M, et al (2014) Antigenic and receptor binding properties of enterovirus 68. J Virol 88: 2374–2384. 24371050 10.1128/JVI.03070-13 PMC3958110

[27] Sabesan S, Bock K, Paulson JC (1991) Conformational analysis of sialyloligosaccharides. Carbohydr Res 218: 27–54. 1802388 10.1016/0008-6215(91)84084-r

[28] Rossmann MG (1989) The canyon hypothesis. Hiding the host cell receptor attachment site on a viral surface from immune surveillance. J Biol Chem 264: 14587–14590. 2670920

[29] Zhou L, Luo Y, Wu Y, Tsao J, Luo M (2000) Sialylation of the host receptor may modulate entry of demyelinating persistent Theiler's virus. J Virol 74: 1477–1485. 10627559 10.1128/jvi.74.3.1477-1485.2000 PMC111483

[30] Fry EE, Newman JW, Curry S, Najjam S, Jackson T, et al (2005) Structure of Foot-and-mouth disease virus serotype A10 61 alone and complexed with oligosaccharide receptor: receptor conservation in the face of antigenic variation. J Gen Virol 86: 1909–1920. 15958669 10.1099/vir.0.80730-0

[31] Guzman-Aranguez A, Argueso P (2010) Structure and biological roles of mucin-type O-glycans at the ocular surface. Ocul Surf 8: 8–17. 20105403 10.1016/s1542-0124(12)70213-6 PMC2847370

[32] Nilsson EC, Storm RJ, Bauer J, Johansson SM, Lookene A, et al (2011) The GD1a glycan is a cellular receptor for adenoviruses causing epidemic keratoconjunctivitis. Nat Med 17: 105–109. 21151139 10.1038/nm.2267

[33] Spjut S, Qian W, Bauer J, Storm R, Frangsmyr L, et al (2011) A potent trivalent sialic acid inhibitor of adenovirus type 37 infection of human corneal cells. Angew Chem Int Ed Engl 50: 6519–6521. 21648036 10.1002/anie.201101559 PMC3210828

[34] Kitov PI, Sadowska JM, Mulvey G, Armstrong GD, Ling H, et al (2000) Shiga-like toxins are neutralized by tailored multivalent carbohydrate ligands. Nature 403: 669–672. 10688205 10.1038/35001095

[35] Diebold Y, Calonge M, Enriquez de Salamanca A, Callejo S, Corrales RM, et al (2003) Characterization of a spontaneously immortalized cell line (IOBA-NHC) from normal human conjunctiva. Invest Ophthalmol Vis Sci 44: 4263–4274. 14507870 10.1167/iovs.03-0560

[36] Kabsch W (2010) Xds. Acta Crystallogr D Biol Crystallogr 66: 125–132. 20124692 10.1107/S0907444909047337 PMC2815665

[37] Collaborative Computational Project N (1994) The CCP4 suite: programs for protein crystallography. Acta Crystallogr D Biol Crystallogr 50: 760–763. 15299374 10.1107/S0907444994003112

[38] Schrodinger LLC (2010) The PyMOL Molecular Graphics System, Version 1.3r1.

[39] Murshudov GN, Vagin AA, Dodson EJ (1997) Refinement of macromolecular structures by the maximum-likelihood method. Acta Crystallogr D Biol Crystallogr 53: 240–255. 15299926 10.1107/S0907444996012255

[40] Adams PD, Afonine PV, Bunkoczi G, Chen VB, Davis IW, et al (2010) PHENIX: a comprehensive Python-based system for macromolecular structure solution. Acta Crystallogr D Biol Crystallogr 66: 213–221. 20124702 10.1107/S0907444909052925 PMC2815670

[41] Emsley P, Lohkamp B, Scott WG, Cowtan K (2010) Features and development of Coot. Acta Crystallogr D Biol Crystallogr 66: 486–501. 20383002 10.1107/S0907444910007493 PMC2852313

[42] Baker NA, Sept D, Joseph S, Holst MJ, McCammon JA (2001) Electrostatics of nanosystems: application to microtubules and the ribosome. Proc Natl Acad Sci U S A 98: 10037–10041. 11517324 10.1073/pnas.181342398 PMC56910

[43] Allinger NL, Rahman M, Lii JH (1990) A molecular mechanics force field (MM3) for alcohols and ethers. Journal of the American Chemical Society 112: 8293–8307.

[44] Morris GM, Goodsell DS, Halliday RS, Huey R, Hart WE, et al (1998) Automated docking using a Lamarckian genetic algorithm and an empirical binding free energy function. Journal of Computational Chemistry 19: 1639–1662.

[45] Krieger E, Darden T, Nabuurs SB, Finkelstein A, Vriend G (2004) Making optimal use of empirical energy functions: Force-field parameterization in crystal space. Proteins: Structure, Function, and Bioinformatics 57: 678–683. 10.1002/prot.20251 15390263

[46] Duan Y, Wu C, Chowdhury S, Lee MC, Xiong G, et al (2003) A point-charge force field for molecular mechanics simulations of proteins based on condensed-phase quantum mechanical calculations. Journal of Computational Chemistry 24: 1999–2012. 14531054 10.1002/jcc.10349

[47] Woods RJ, Dwek RA, Edge CJ, Fraser-Reid B (1995) Molecular Mechanical and Molecular Dynamic Simulations of Glycoproteins and Oligosaccharides. 1. GLYCAM_93 Parameter Development. The Journal of Physical Chemistry 99: 3832–3846.

[48] Gouet P, Courcelle E, Stuart DI, Metoz F (1999) ESPript: analysis of multiple sequence alignments in PostScript. Bioinformatics 15: 305–308. 10320398 10.1093/bioinformatics/15.4.305

