# Supplementary figures and images for: A Sialic Acid Binding Site in a Human Picornavirus
Source: PLoS Pathog. 2014 Oct 16;10(10):e1004401. doi: 10.1371/journal.ppat.1004401 (PMC4199766; doi:10.1371/journal.ppat.1004401)

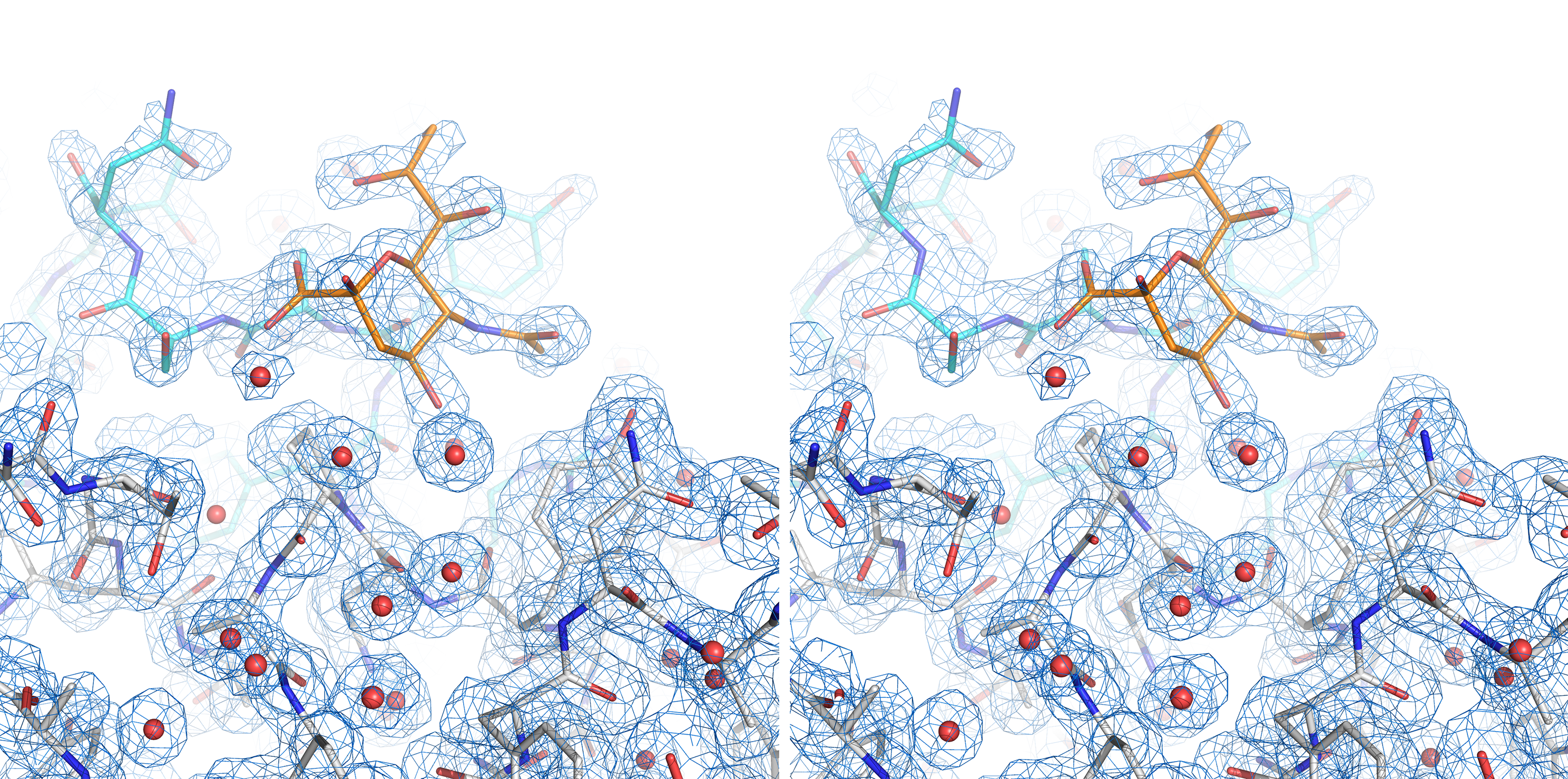

Supplement: Figure S1 — Stereo representation of the electron density around the sialic acid binding site. A (2Fo-Fc)-electron density map around the glycan binding site is shown in a stereo representation to elucidate the quality of the refined CVA24v-6SL model. The sialic acid entity (light orange), the VP1 protomer (grey) and the clockwise rotated VP1 protomer (cyan) are shown in a stick representation. Water molecules (red) are shown as spheres. Some residues (727-730) in the DE-loop of VP1 showed a higher flexibility (not shown) which is reflected in higher B-Factors (26 Å2 compared to the mean of 13 Å2 for all protein atoms). (TIF) [file ppat.1004401.s001.tif]

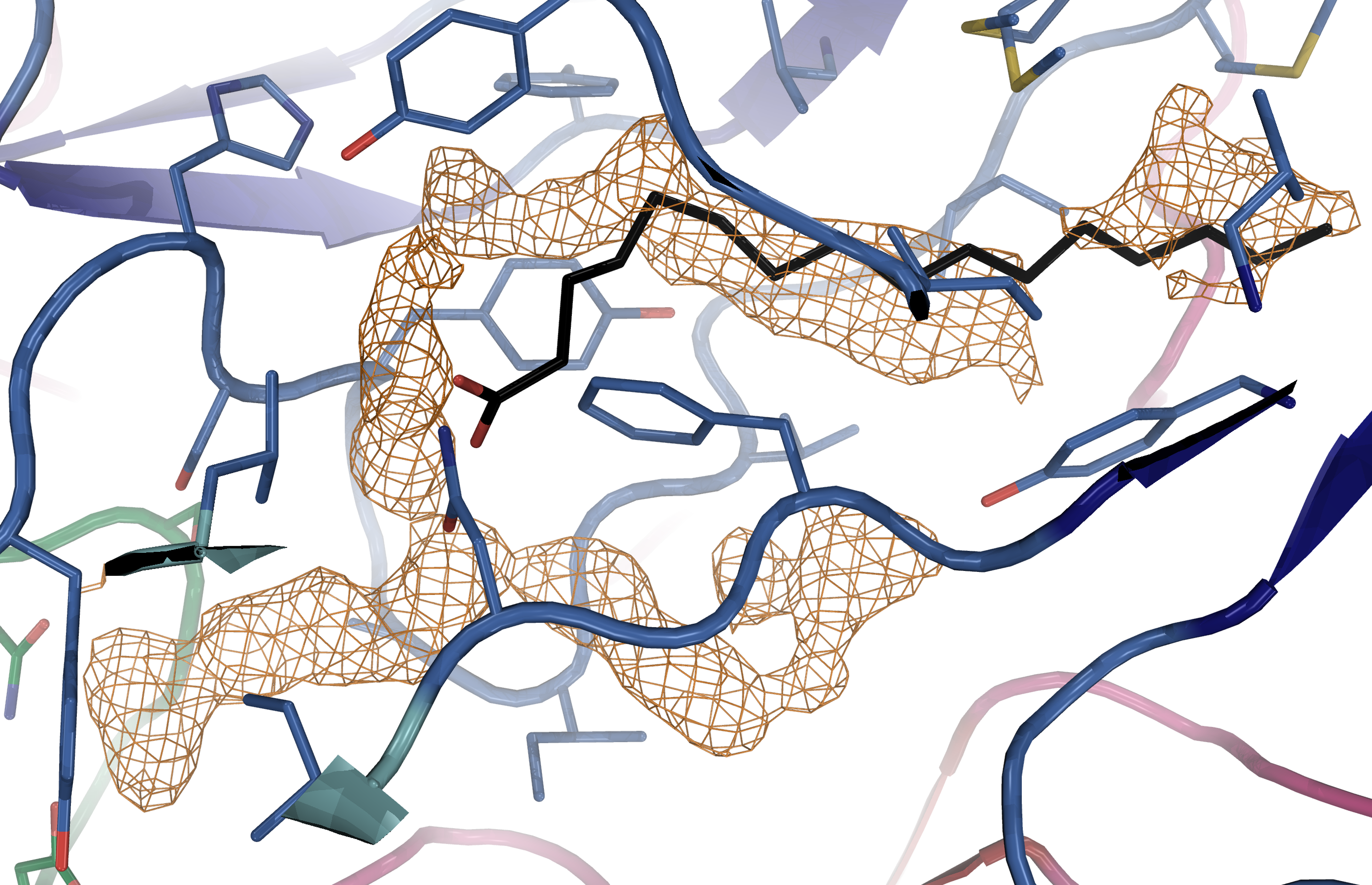

Supplement: Figure S2 — Electron density for the pocket factor in CVA24v. The protomers VP1 (blue), VP2 (green) and VP3 (red) are shown around the pocket factor cavity. The pocket factor, a hydrophobic compound bound in a cavity on the bottom of the canyon of the virus [22] is important for virus stabilization and is released during the attachment of immunoglobulin-like receptors at the canyon to facilitated RNA release. We found substantial positive (Fo-Fc)-electron density (orange, σ-level of 2.9) in this cavity of CVA24v for a branched ligand. A ceramide would in principle fit into the electron density but could not unambiguously identify as a pocket factor. A superimposition with coxsackievirus B3 (CVB3) (4GB3, black) revealed differences in the pocket that would exclude the binding of a long fatty acid as observed for this strain. (TIF) [file ppat.1004401.s002.tif]

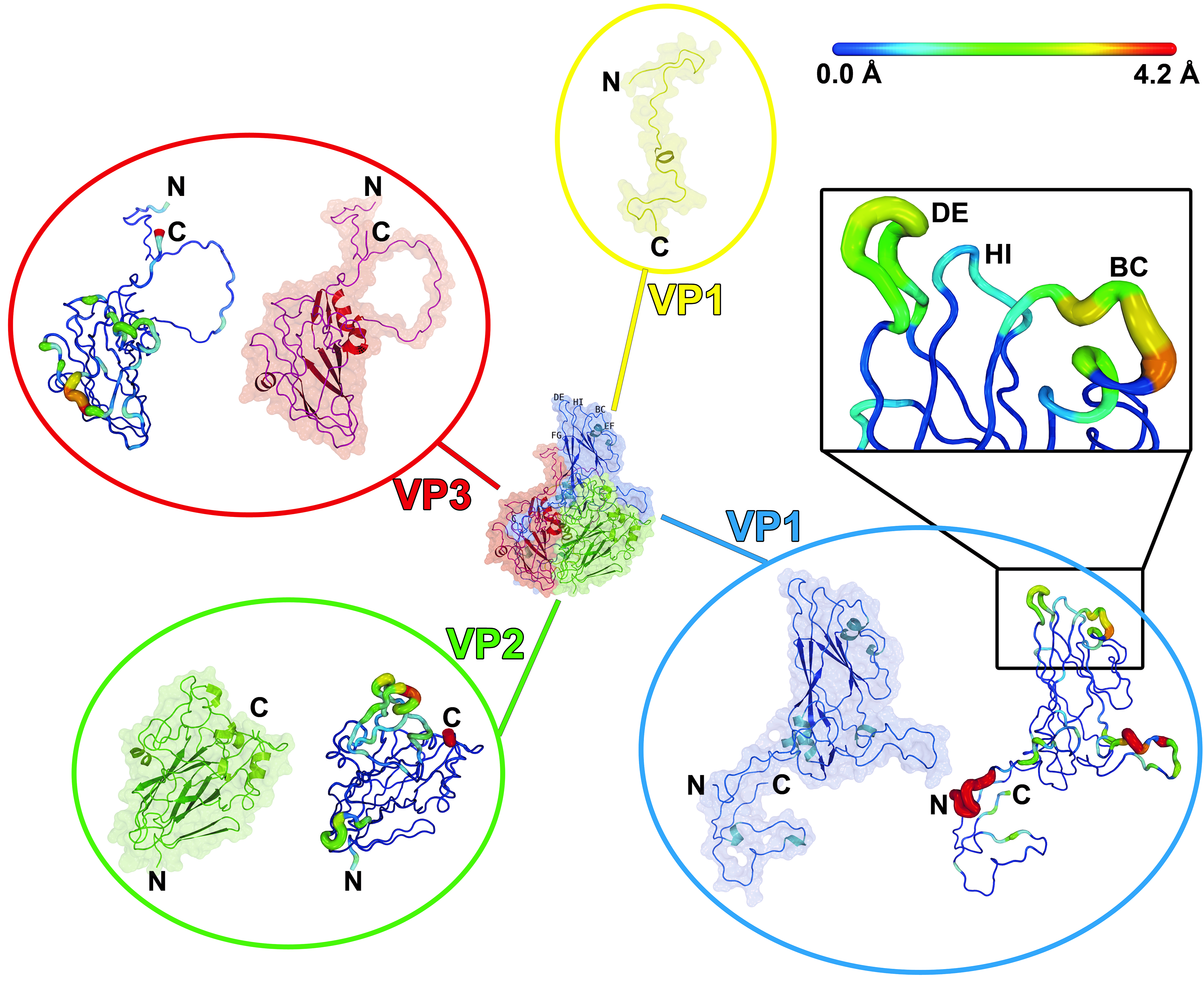

Supplement: Figure S3 — Assembly of the capsid proteins and structure comparison. The capsid proteins VP1 (blue), VP2 (green), VP3 (red), and VP4 (yellow) are shown as cartoon and as surface representation from the identical orientation. The termini of the protein chain are labeled. Moreover, the capsid proteins VP1-3 were colored by the mean Cα rms deviation of ten virus capsid proteins listed in table S2 from blue (zero rms deviation) to red (4.2 Å rms deviation). A close-up of the glycan binding region (black box) revealed the largest structural differences in the DE- and BC- loop. (TIF) [file ppat.1004401.s003.tif]

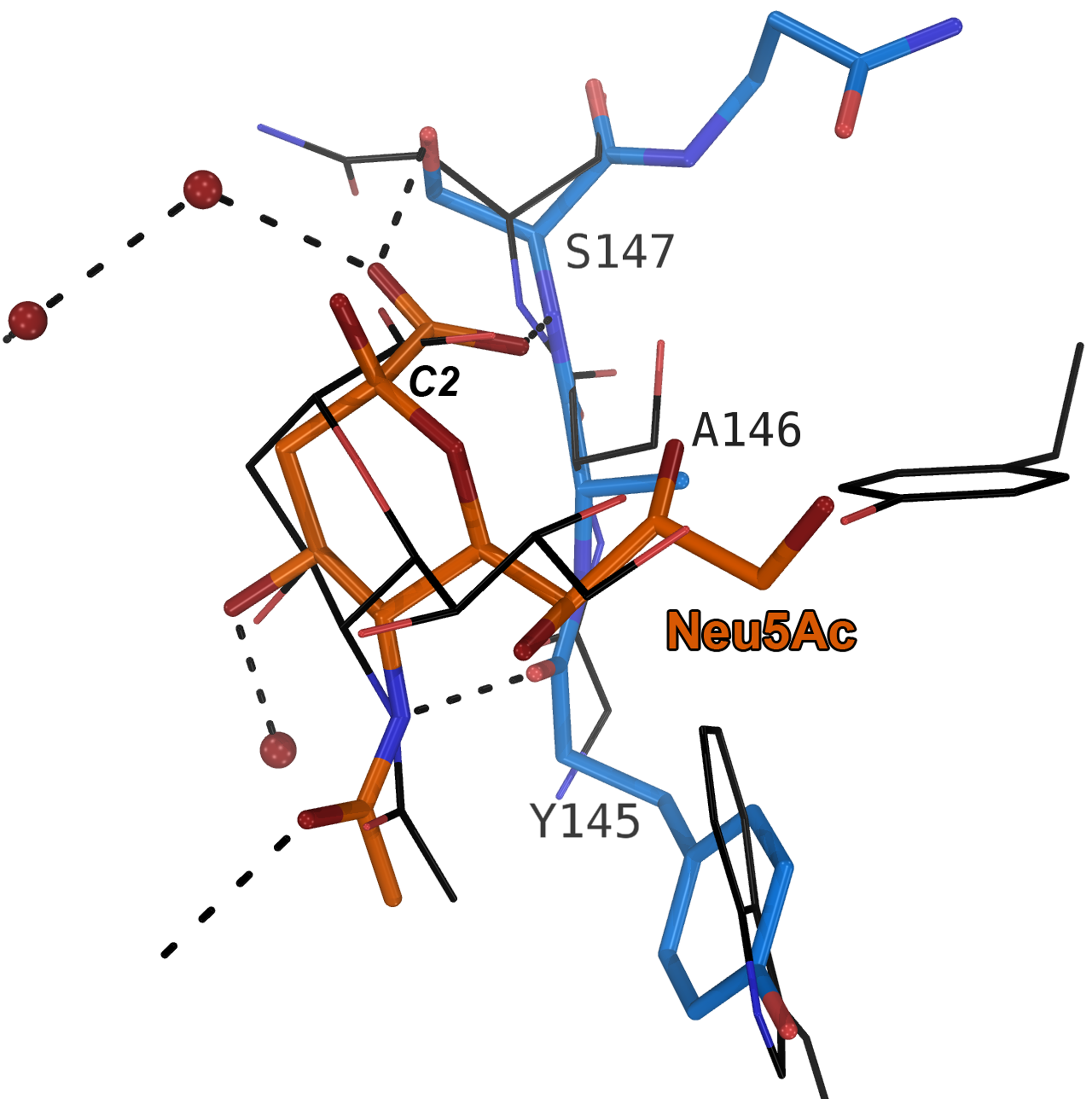

Supplement: Figure S4 — Similarities in sialic acid recognition between CVA24v and influenza virus hemagglutinin. A superposition of the sialic acid-binding regions is shown, with the sialic acids colored orange for CVA24v and black for influenza hemagglutinin (pdb-code: 1HGG). The main chain interaction of CVA24v and influenza virus hemagglutinin to Neu5Ac are generally similar, although serine interacts with the carboxy moiety from the opposite side. (TIF) [file ppat.1004401.s004.tif]

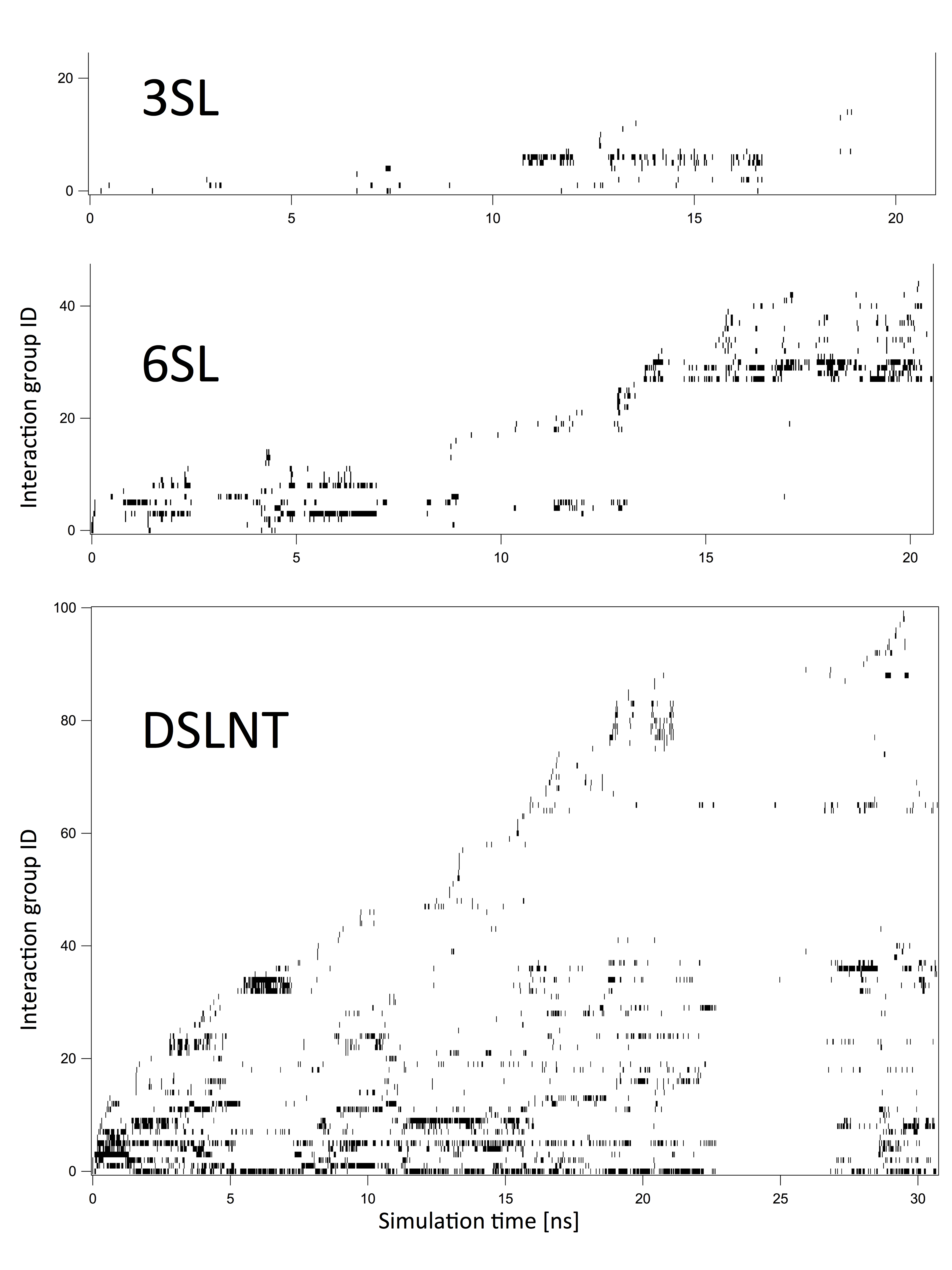

Supplement: Figure S5 — Trajectories of favourable intermolecular contacts. Analysis of favourable (hydrophopic and H-bond) intermolecular atom contacts using Conformational Analysis Tools (www.md-simulations.de/CAT/). The molecular dynamics simulation was performed with the virus pentamer in complex with five ligands in explicit solvent. Interaction group trajectories are shown for one selected ligand only. A new interaction group (atom pair) is allocated if one of the following conditions were met: C-C distance <4.0 Å (hydrophobic) or H-bond donor-acceptor distance <3.5 Å. The Neu5Ac residue located in the crystallographically determined binding site was excluded from the analysis. It can be seen that the number of possible additional contacts increases in the sequence 3SL<6SL<DSLNT and that contacts are generally transient. (TIF) [file ppat.1004401.s005.tif]

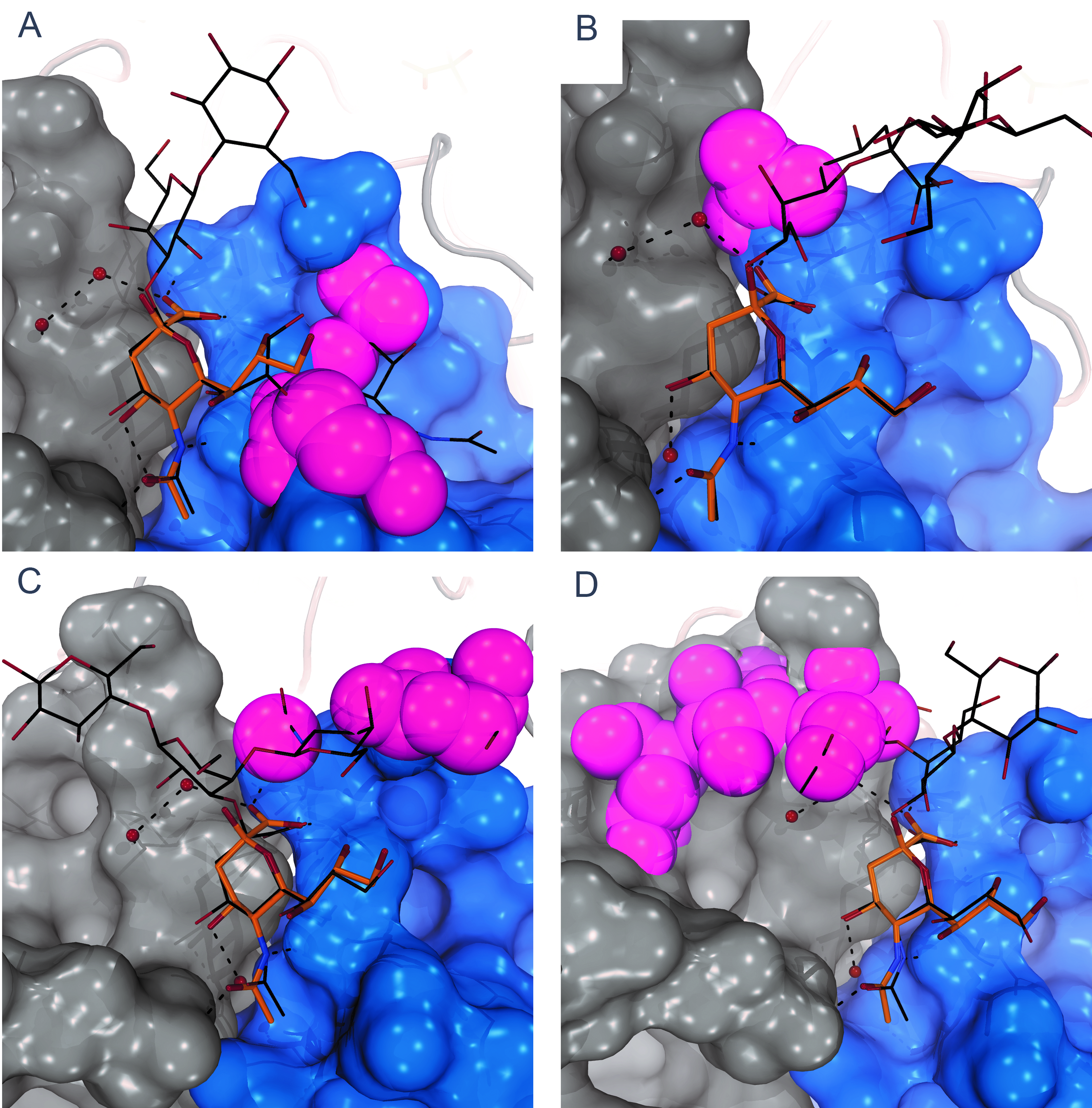

Supplement: Figure S6 — Sterical clashes of glycans superposed to the Neu5Ac entity in CVA24v. α2,8-Neu5Ac-α2,3-Neu5Ac linked glycans are unlikely to bind to CVA24v as binding would result in a collision with the protein skeleton (shown as pink spheres) independently of whether binding occurs with the bridged Neu5Ac entity (A) or terminal sialic acid entity (B). Moreover, steric restriction hampered binding of glycans with a β-branch in respect to the Neu5Ac moiety, as shown for GM1 (C) or GD1a (D). (TIF) [file ppat.1004401.s006.tif]

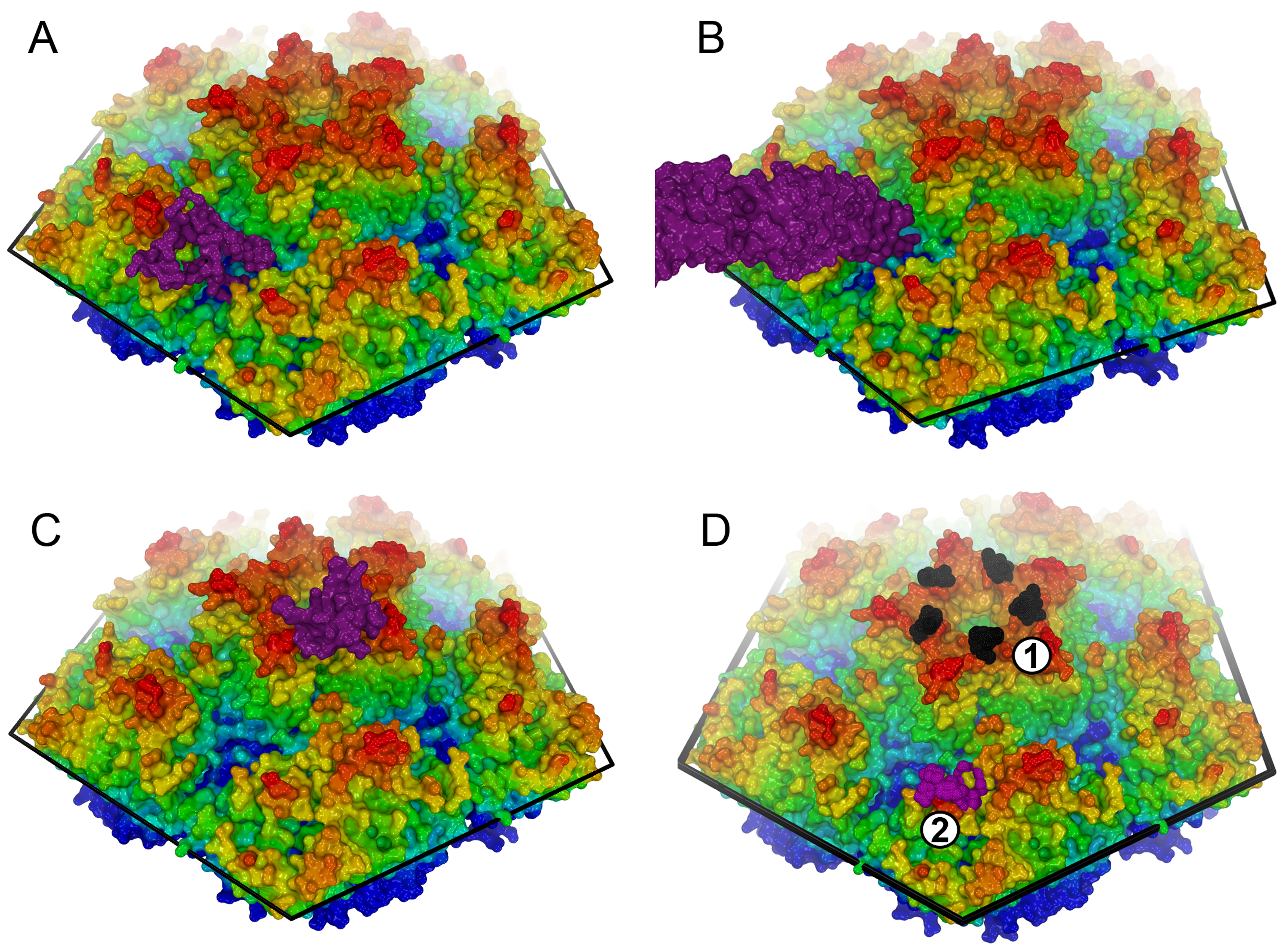

Supplement: Figure S7 — Location of picornavirus receptors. Receptors (purple) of the immunoglobulin superfamily bind into the “canyon” of the picornavirus capsid as shown for coxsackievirus B3 in complex with CAR (A) (adopted from pdb 1JEW [15]) or coxsackievirus A21 in complex ICAM-1 (B) (adopted from pdb 1Z7Z [13]). Receptors that do not belong to this superfamily do bind elsewhere, e.g. Rhinovirus 2 in complex with the LDL receptor (C) (adopted from pdb 1V9U [14]) or glycan receptors (D) such as the oligosaccharide receptor (purple, labeled as 2) of Foot-and-mouth disease virus serotype A1061 (adopted from pdb 1ZBA [30]). The binding site of the glycan receptor (black) of CVA24v (labeled as 1) is located at the LDL receptor binding site of Rhinovirus 2. (TIF) [file ppat.1004401.s007.tif]

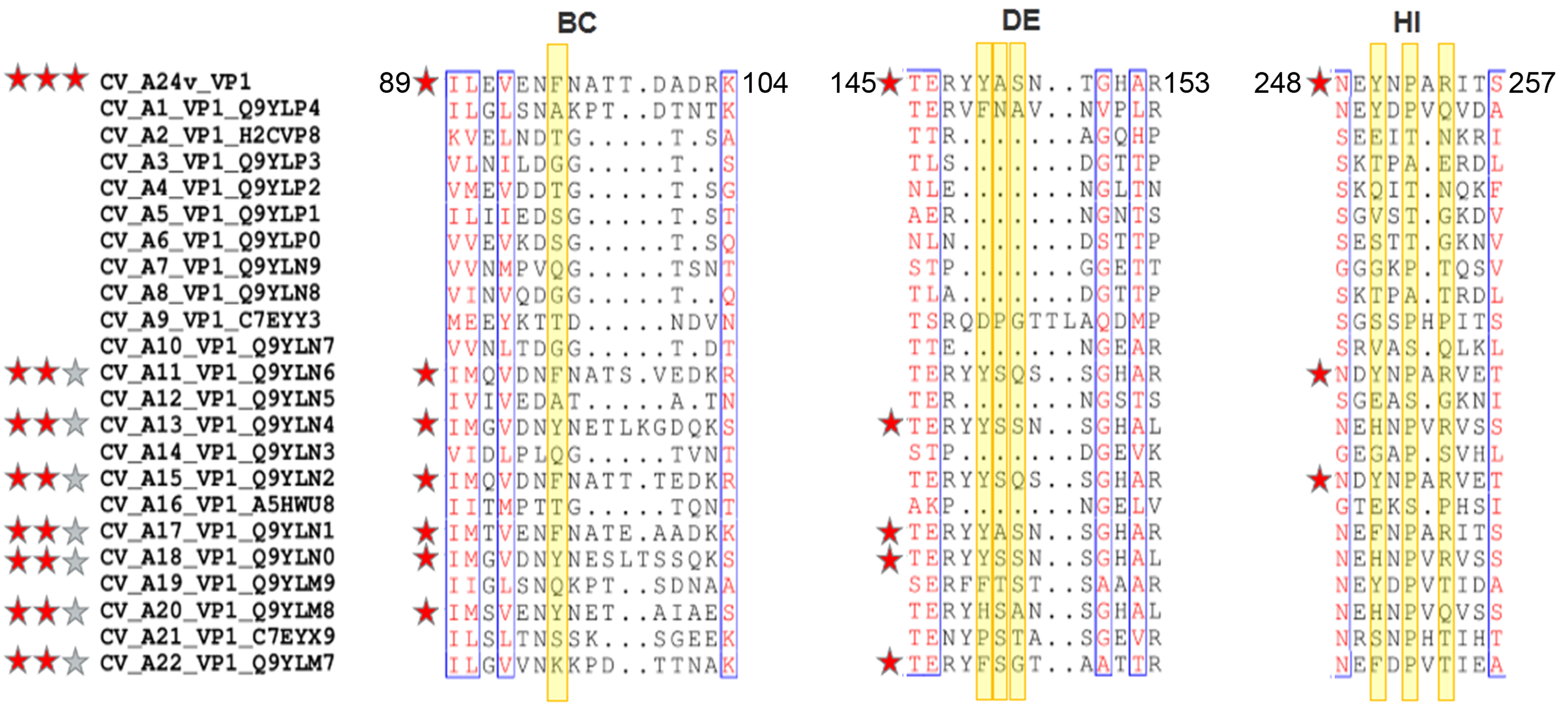

Supplement: Figure S8 — The unique binding site of CVA24v. Important residues for Neu5Ac binding (marked yellow) are found on the BC-, DE-, and the HI-loop. We suggest that sialic acid binds (three red stars) only if the residues are functionally conserved in all three loop regions. A residue is concerned as functionally conserved, if its side chain is capable to perform a similar interaction, e.g. hydrogen bond compared to CVA24v. A sequence comparison revealed unique character of the glycan binding site of CVA24v as compared to other coxsackieviruses in species Enterovirus A, B, and C. (TIF) [file ppat.1004401.s008.tif]

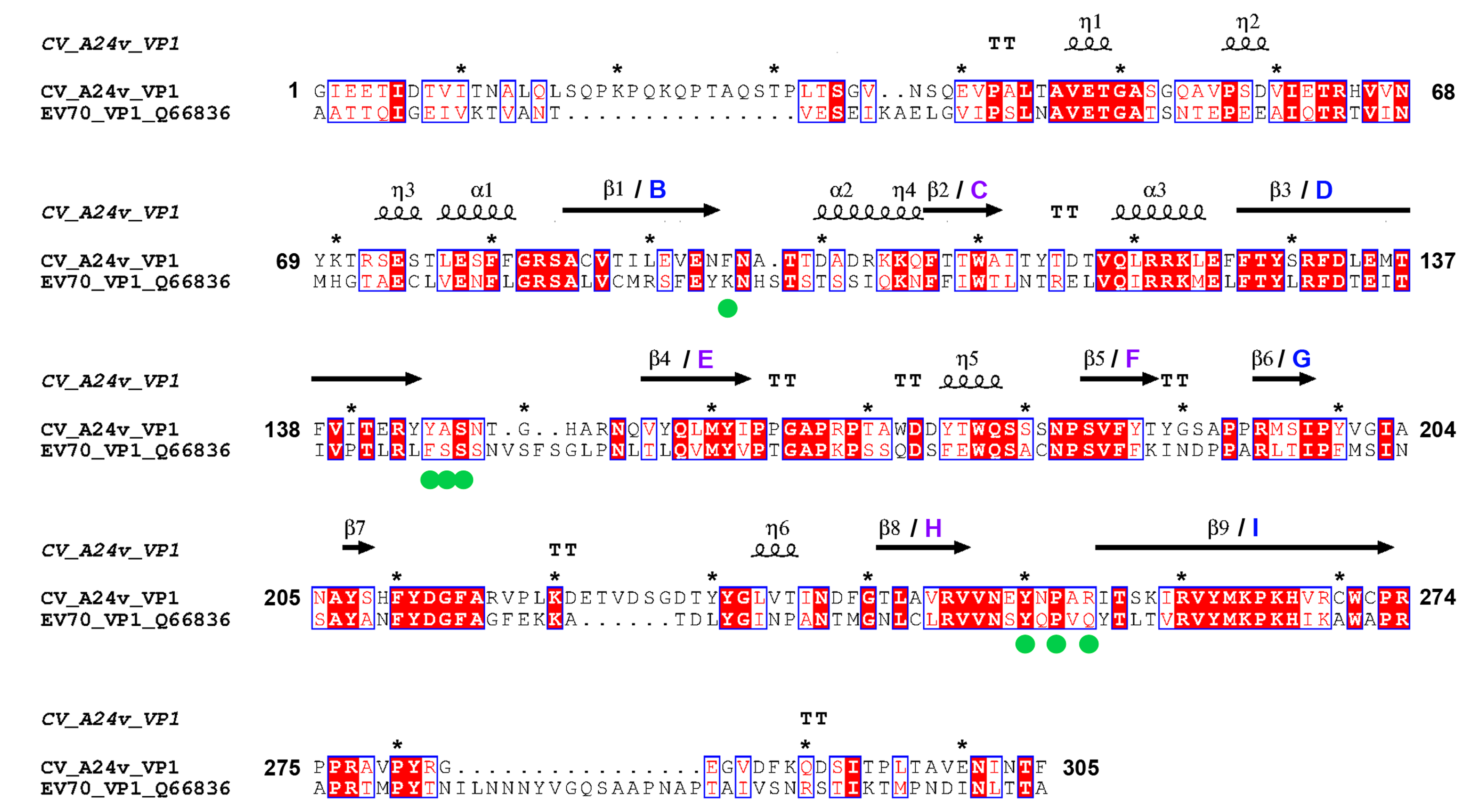

Supplement: Figure S9 — Sequence comparison of the AHC-causing viruses CVA24v and EV70. This figure was modified from ESPRIPT [48] output. α-helices, 310-helices and π-helices are displayed as squiggles. The η and α symbol refers to a 310-helix and α-helix, respectively. β-strands are rendered as arrows, strict β-turns as TT letters and strict α-turns as TTT. Strictly conserved residues are marked by a red box and similar residues are shown as red character. Positions of CVA24v involved in glycan recognition are marked by green spheres. The β-strands of the jelly roll motif are labeled from “B to I” in agreement with the jelly roll nomenclature. (TIF) [file ppat.1004401.s009.tif]
