# Supplementary material for: A Sialic Acid Binding Site in a Human Picornavirus
Source: PLoS Pathog. 2014 Oct 16;10(10):e1004401. doi: 10.1371/journal.ppat.1004401 (PMC4199766; doi:10.1371/journal.ppat.1004401)
Supplement: Table S1 — Structurally related capsid proteins of VP1-3.a Results of the structure comparison of VP1, VP2 and VP3 to ten different homologous picornavirus capsids using DALI [23]. (PDF) [file ppat.1004401.s010.pdf]

Table S1. Structurally related capsid proteins of VP1-3[a]

Virus	PDB entry	Capsid protein	Z-Score	Sequence identity [%]	Cα-rmsd [Å]	# of aligned residues
Coxsackie Virus A21,	1z7s	VP1	28.9	76	1.3	278 / 283
(3.2 Å)		VP2	42.3	73	0.7	263 / 267
		VP3	31.4	86	0.4	233 / 239
Poliovirus Type 3,	1pvc	VP1	28.2	64	1.2	275 /279
Sabin strain,		VP2	42.1	74	0.7	262 / 266
(2.4 Å)		VP3	31.0	71	0.6	233 / 235
Poliovirus Type 1,	1ar6	VP1	27.9	64	1.3	276 / 283
Mahoney strain,		VP2	42.1	73	0.7	263 / 268
(2.9 Å)		VP3	31.2	73	0.5	233 / 275
Poliovirus Type 2,	1eah	VP1	27.0	65	1.3	269 / 272
Lansing strain,		VP2	41.9	73	0.6	260 / 262
(2.9 Å)		VP3	31.0	71	0.5	233 /235
Swine vesicular disease	1oop	VP1	26.6	50	1.2	264 / 271
virus (SVDV),		VP2	37.4	55	1.4	252 / 252
(3.0 Å)		VP3	29.7	59	0.8	233 / 238
Rhinovirus B14,	1ncq	VP1	26.2	43	1.5	269 / 273
(2.5 Å)		VP2	39.0	54	1.5	254 / 255
		VP3	28.3	46	1.0	231 / 236
Coxsackievirus A9,	1d4m	VP1	25.9	50	1.4	265 / 284
(2.9 Å)		VP2	37.3	57	1.3	252 / 252
		VP3	29.0	61	1.1	233 / 238
Coxsackievirus B3,	1cov	VP1	25.9	51	1.3	269 / 273
(3.5 Å)		VP2	37.5	58	1.4	255 / 256
		VP3	29.6	61	0.8	233 / 238
Echovirus 11,	1h8t	VP1	25.8	48	1.3	265 / 290
(2.9 Å)		VP2	37.4	56	1.3	252 / 252
		VP3	29.5	60	0.8	233 / 238
Echovirus 7,	2x5i	VP1	25.8	47	1.4	265 / 277
(3.1 Å)		VP2	37.6	57	1.2	252 / 252
		VP3	29.5	56	0.7	233 / 238

[a] Structure similarity search was performed using DALI[1] . The first ten non-redundant virus structures based on the similarity to VP1 are listed.
