# Supplementary material for: A Sialic Acid Binding Site in a Human Picornavirus
Source: PLoS Pathog. 2014 Oct 16;10(10):e1004401. doi: 10.1371/journal.ppat.1004401 (PMC4199766; doi:10.1371/journal.ppat.1004401)
Supplement: Table S2 — Structurally related capsid proteins of VP4.a Results of the structure comparison of VP4 to homologous picornavirus capsids using DALI [23]. (PDF) [file ppat.1004401.s011.pdf]

Table S2. Structurally related capsid proteins of VP4[a]

Virus	PDB entry	Capsid protein	Z-Score	Sequence identity [%]	Cα-rmsd [Å]	# of aligned residues
Rhinovirus B3, (3.0 Å)	1rhi	VP4	4.0	70	0.7	43 / 43
Rhinovirus B14, (2.5 Å)	1ncq	VP4	3.6	68	0.7	40 / 40
Enterovirus 71 (2.6 Å)	3vbf	VP4	2.4	51	1.1	39 / 58

[a] Structure similarity search was performed using DALI[1]. Redundant entries were removed.
